# Distribution of Extracellular Glutamate in the Neuropil of Hippocampus

**DOI:** 10.1371/journal.pone.0026501

**Published:** 2011-11-01

**Authors:** Melissa A. Herman, Ben Nahir, Craig E. Jahr

**Affiliations:** Vollum Institute, Oregon Health and Science University, Portland, Oregon, United States of America; Federal University of Rio de Janeiro, Brazil

## Abstract

Reported values of extracellular glutamate concentrations in the resting state depend on the method of measurement and vary ∼1000-fold. As glutamate levels in the micromolar range can cause receptor desensitization and excitotoxicity, and thus affect neuronal excitability, an accurate determination of ambient glutamate is important. Part of the variability of previous measurements may have resulted from the sampling of glutamate in different extracellular compartments, e.g., synaptic versus extrasynaptic volumes. A steep concentration gradient of glutamate between these two compartments could be maintained, for example, by high densities of glutamate transporters arrayed at the edges of synapses. We have used two photon laser scanning microscopy and electrophysiology to investigate whether extracellular glutamate is compartmentalized in acute hippocampal slices. Pharmacological blockade of NMDARs had no effect on Ca^2+^ transients generated in dendritic shafts or spines of CA1 pyramidal neurons by depolarization, suggesting that ambient glutamate is too low to activate a significant number of NMDARs. Furthermore, blockade of transporters did not flood the synapse with glutamate, indicating that synaptic NMDARs are not protected from high concentrations of extrasynaptic glutamate. We suggest that, in the CA1 region of hippocampus, glutamate transporters do not create a privileged space within the synapse but rather keep ambient glutamate at very low levels throughout the neuropil.

## Introduction

The excitatory neurotransmitter glutamate is not degraded in the extracellular space following release. Rather, clearance of glutamate release into the synaptic cleft depends on diffusion and uptake to terminate synaptic transmission [1]–[6] and prevent excitotoxicity [7]. Despite the efficiency and high expression density of glutamate transporters [8], [9], a measurable concentration of glutamate exists in the extracellular space of neuronal tissue. Estimates of this ambient concentration range from tens of nanomolar to tens of micromolar depending on the measurement technique used; electrophysiological methods yield lower estimates than microdialysis or amperometry [10]–[19].

We estimated in a previous study that extracellular glutamate in acute hippocampal slices is ∼25 nM, a concentration that produces a small but detectable tonic current in CA1 pyramidal neurons that is mediated by N-methyl-D-aspartic acid receptors [12] (NMDARs). This current represents the activity of all NMDARs expressed by the neuron and does not differentiate between synaptic and extrasynaptic receptors. Due to the complex architecture of the neuropil and the heterogeneous distribution of glutamate transporters [9], [20], it has been suggested that ambient glutamate concentrations are much higher in the extrasynaptic space than in the synaptic cleft [21]–[23] giving rise to preferential activation of extrasynaptic NMDARs [13], [24]. As synaptic NMDARs greatly outnumber extrasynaptic NMDARs [25], [26], the 25 nM concentration estimate yielded by our previous approach [12] may mainly reflect the concentration within the cleft, thus dramatically underestimating the glutamate concentration in the extrasynaptic space. In this scenario, the higher estimates of ambient glutamate obtained with microdialysis and amperometry [16]–[19] would reflect measurements of the extrasynaptic space.

To determine the location of NMDARs activated by ambient glutamate, a technique with spatial resolution is required. We have used two photon laser scanning microscopy (2PLSM) and electrophysiology to determine whether a steep concentration gradient exists by measuring Ca^2+^ transients in dendritic shafts and spines mediated by NMDARs. We find that there is not a steep concentration gradient of glutamate between the synaptic and extrasynaptic space and, consequently, that the synaptic compartment is not preferentially shielded by glutamate transporters. We conclude that ambient glutamate is not significantly compartmentalized but rather is universally low throughout the neuropil of the hippocampus.

## Results

**Figure 1:**
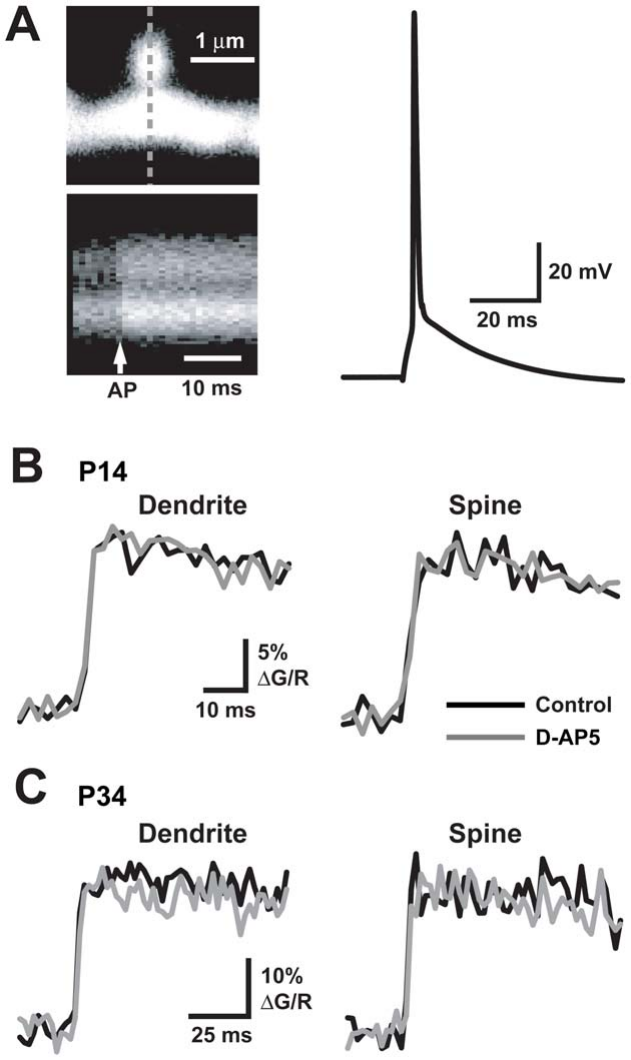
NMDAR antagonism has no differential effect on bAP-evoked Ca^2+^ signals in dendrites or spines. A. Top left: 2PLSM image of a spine and dendrite with dashed line indicating line scan position. Bottom left: During a 500 Hz line scan through spine and dendrite, a bAP (arrow) evokes a Ca^2+^ transient in both compartments. Right: Somatic AP evoked by current injection. B, C. Average Ca^2+^ transients evoked by a bAP measured in the dendrite (left) and spine (right) from a P14 animal (B; n = 11 for both structures) and a P34 animal (C; spine, n = 17; dendrite, n = 13) in the presence and absence of D-AP5 (10–50 µM).

Whole cell current clamp recordings were made from CA1 pyramidal neurons in acute hippocampal slices. The cells were filled through the patch pipette with the morphological dye Alexa Fluor 594 (15 µM) and the Ca^2+^ indicator Fluo-5F (300 µM). To determine if ambient glutamate levels are high enough to bind significant numbers of NMDARs, we measured Ca^2+^ transients in both spines and dendritic shafts evoked by back-propagating action potentials [27] (bAP; Fig. 1). As NMDARs are expressed synaptically and extrasynaptically [4], [28], [29], Ca^2+^ transients evoked by bAPs in the two cellular compartments may be mediated by synaptic and extrasynaptic NMDARs bound by ambient glutamate as well as by VGCCs. As has been shown previously [27], however, pharmacological block of NMDARs did not alter the Ca^2+^ transients in either compartment (Fig. 1B; spine: 88.0±6.90%, dendrite: 90.5±5.71%; p>0.1; n = 11). Though NMDAR activation by exogenous glutamate boosts bAP-elicited Ca^2+^ transients [30], the present results, and those of others [27], suggest that there is little tonic activation of NMDARs in either spines or dendritic shafts. Furthermore, there appears to be no developmental shift in the tonic activation of NMDARs since D-AP5 also failed to alter the Ca^2+^ transients from spines (Fig. 1C; 94.4±5.27%; p>0.3; n = 17) and dendrites (Fig. 1C; 90.7±4.78%; p>0.05; n = 13) of CA1 pyramidal neurons from older animals (P33–40).

**Figure 2:**
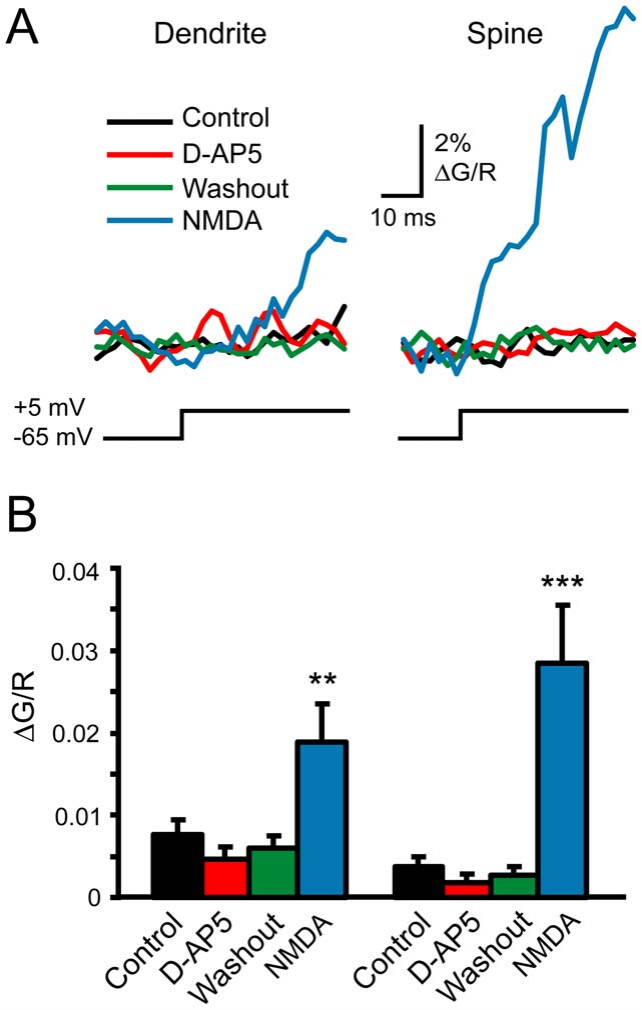
Ambient glutamate concentrations are too low to generate significant Ca^2+^ influx through NMDARs in dendrites or spines. A. Averaged Ca^2+^ transients (500 Hz line scans) evoked by 40 ms voltage step in a dendrite (left) and spine (right) in control (black), D-AP5 (red, 10 µM), after a 10 min washout of D-AP5 (green), and in 5 µM NMDA (blue). Mibefradil (20 µM), nimodipine (20 µM) and TTX (0.5 µM) were present throughout. B. Comparison of Ca^2+^ transient amplitudes in each condition (n = 11). Error bars indicate standard error of the mean (SEM). Significance determined by Friedman ANOVA with Conover posthoc test: **p<0.01; ***p<0.001.

This experiment may not be sensitive enough to detect low level activation of NMDARs because of infrequent channel gating and because bAPs may be too short to engage the slow components of NMDAR Mg^2+^ unblock [31]. In addition, Ca^2+^ influx through infrequently open NMDARs during a bAP may be small relative to the Ca^2+^ contribution from VGCCs. To increase the potential contribution of NMDARs to the Ca^2+^ transient, pyramidal cells were voltage clamped at −65 mV and stepped to +5 mV for 40 ms (Fig. 2A) in the presence of mibefradil and nimodipine (both at 20 µM), antagonists of the predominant VGCCs on pyramidal cell dendrites and spines [32], along with TTX (0.5 µM). Subsequent application of D-AP5 did not affect the voltage step-evoked Ca^2+^ transient (Fig. 2B; p>0.1 for both spine and dendrite). To ensure that this technique was sensitive enough to detect NMDAR activation, we applied 5 µM NMDA to the superfusate (equivalent to ∼250 nM glutamate) [12], [33] following washout of D-AP5. NMDA significantly increased the voltage step-evoked Ca^2+^ signal (Fig. 2; spine: 7.54 fold increase, p<0.001; dendrite: 2.46 fold increase, p<0.01; n = 11). Data from both apical and basal dendrites were pooled since no differences were observed between these two regions. Taken together, these data reinforce the notion that ambient glutamate is maintained at low concentrations, producing minimal NMDAR activation in both synaptic and extrasynaptic compartments.

**Figure 3:**
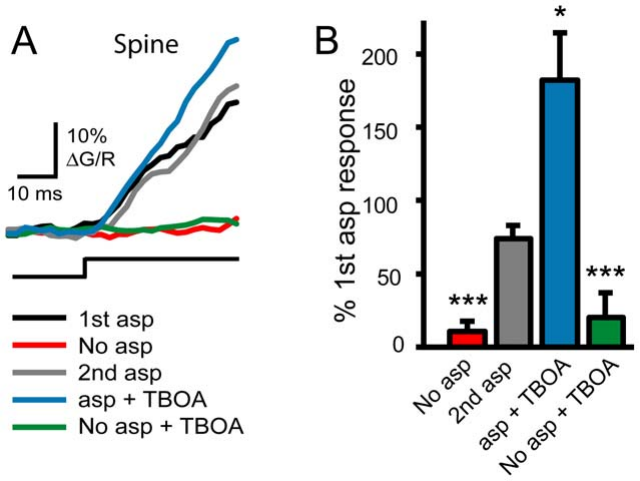
Transporter blockade does not reveal an ambient glutamate concentration gradient between extracellular compartments. A. Average Ca^2+^ increase in a spine during a 40 ms voltage step, with iontophoresis of L-aspartate (black), without iontophoresis (red), a second L-aspartate application (gray), L-aspartate in the presence of 100 µM TBOA (blue), and TBOA alone (green). B. Comparison of spine Ca^2+^ transients in each condition, normalized to the first response to L-aspartate iontophoresis (n = 5). Error bars indicate SEM. Significance determined by Friedman ANOVA with Conover posthoc test: *p<0.05; **p<0.01; ***p<0.001.

We approached the issue of transporter distribution and preferential synaptic protection by blocking glutamate uptake. If the extrasynaptic glutamate concentration is higher than that in the cleft because transporters prevent diffusion of glutamate into the synapse, blocking transporters should result in a large Ca^2+^ increase in the spine as extrasynaptic glutamate rushes into the cleft and activates synaptic NMDARs. Spines exhibited a Ca^2+^ increase during a 40 ms depolarization with iontophoresis of the glutamate transporter substrate and NMDAR agonist, L-aspartate (Fig. 3A; black and gray traces), confirming the presence of NMDARs. However, TBOA (100 µM) did not increase the Ca^2+^ transient in the same spines during the 40 ms depolarization when compared to the control voltage step without L-aspartate iontophoresis (Fig. 3, compare green and red traces; 20.6±13.62%; p>0.5; n = 5;). TBOA was effective in blocking transporters, however, as the NMDAR-mediated Ca^2+^ signal evoked by iontophoresis of L-aspartate was increased in the presence of TBOA (Fig. 3). This result indicates that glutamate transporters do not normally generate a concentration gradient of ambient glutamate between extrasynaptic and synaptic extracellular compartments.

## Discussion

Estimates of the average extracellular glutamate concentration range from ∼25 nM to up to ∼30 µM. Based on electrophysiological measurements of receptor activation, ambient glutamate levels are very low [10], [12]–[15], [34] whereas microdialysis [16]–[18] and amperometry [19] report much higher levels. The various techniques may measure glutamate in different extracellular compartments such that, for example, NMDAR-mediated currents mainly report synaptic glutamate levels whereas microdialysis and amperometry measure extrasynaptic glutamate. Indeed, the distribution of ambient glutamate within the extracellular space is an issue of debate [21], [23] and a steep concentration gradient between extrasynaptic and synaptic regions of the neuropil has been proposed [23]. We report that in area CA1 of the hippocampus, however, low ambient glutamate concentrations are maintained throughout the neuropil. Any differences in glutamate concentrations across the neuropil must be quite modest.

We observed NMDAR-mediated Ca^2+^ elevations in the dendrite and spine only in response to exogenous application of NMDAR agonists, suggesting NMDARs on both structures are rarely bound by ambient glutamate. In whole cell recordings, a small NMDAR-mediated current is activated by ambient glutamate and is increased by inhibiting transport [10]–[13]. However, this tonic current represents the activity of only a small fraction of the total number of NMDARs expressed by a neuron. Detection of such a small fractional activation in a single spine, which expresses at least 1000-fold fewer NMDARs than the whole cell, would be unlikely, despite the sensitivity of 2PLSM [35], [36].

Detecting ambient glutamate in the extrasynaptic space using NMDAR Ca^2+^ influx may be problematic if the expression of these receptors is low in this compartment [25], [26] and if the dendritic signal is contaminated by bound calcium indicator diffusing from activated spines [37]. Such contamination could also result in slower or delayed Ca^2+^ signals in dendritic shafts. As an alternative test for high extrasynaptic glutamate concentrations, we monitored Ca^2+^ in spines while blocking glutamate transporters. This should collapse any existing extracellular glutamate gradient and allow synaptic NMDARs [38] to respond to extrasynaptic levels of glutamate. Because the synaptic cleft volume is small, relative to the volume of the extrasynaptic space, extracellular glutamate in the synapse will rapidly approach the concentration in the extrasynaptic compartment once the gradient is disrupted. Therefore, if extrasynaptic levels are in the micromolar range, transporter block should cause large Ca^2+^ elevations in spines. However, TBOA did not increase the spine Ca^2+^ signal. As 5 µM NMDA activates large NMDAR-mediated Ca^2+^ transients in spines, the ineffectiveness of TBOA suggests that extrasynaptic levels of glutamate must be substantially lower than 250 nM, similar to that normally present in the quiescent cleft.

Quantitative immuno-EM studies report a higher number of transporters on astrocyte membranes facing synapse-rich neuropil than facing non-synaptic structures or other astrocyte processes [9] suggesting that ambient glutamate levels could be heterogeneously distributed. However, in stratum radiatum transporter density decreases only two-fold, from ∼10,000 to ∼5,000 per µm^2^ of astrocyte membrane. Using this distribution of transporters, models of the extracellular space predict that the glutamate concentration is in the range of 30–50 nM throughout the neuropil of hippocampus [14], similar to previous experimental estimates [10], [12], [13], [34]. In addition, EM studies indicate that astrocytic processes thread throughout the neuropil of hippocampal stratum radiatum, associating both with synaptic and non-synaptic components of pyramidal neurons, but rarely completely encase synapses [39], [40]. Together with our present findings, these studies indicate that neither spatially heterogeneous transporter expression nor glial investiture of synapses is sufficient to result in compartmentalization of ambient glutamate in stratum radiatum. Instead, extracellular glutamate levels appear to be universally low, except immediately following release.

## Materials and Methods

### Slice preparation

Sprague-Dawley rats (P14–40) were deeply anesthetized with isoflurane and decapitated in compliance with the Oregon Health & Science University Institutional Animal Care and Use Committee approved protocol. Hippocampi were isolated, and transverse slices were cut (300 µm) on a vibroslicer (Leica) in an ice-cold solution containing (in mM): 110 choline chloride, 7 MgCl_2_, 2.5 KCl, 1.25 NaH_2_PO_4_, 0.5 CaCl_2_, 1.3 Na-ascorbate, 25 NaHCO_3_, and 10 glucose (saturated with 95% O_2_/5% CO_2_). Slices were transferred to an incubation chamber containing the following extracellular solution (in mM): 119 NaCl, 2.5 KCl, 2.0 CaCl_2_, 1.3 MgCl_2_, 1.0 NaH_2_PO_4_, 26.2 NaHCO_3_, and 11 glucose (saturated with 95% O_2_/5% CO_2_). Slices were incubated at 34°C for 30–45 minutes then maintained at room temperature. For older animals (P33–40), 100 µM kynurenate was added to the cutting solution and 1.3 mM Na-ascorbate to the extracellular recording solution.

### Experimental Procedures

Whole cell recordings were obtained from CA1 pyramidal neurons visually identified with infrared contrast optics [41]. D-serine (10 µM), 2,3-Dioxo-6-nitro-1,2,3,4-tetrahydrobenzo[*f*]quinoxaline-7-sulfonamide (NBQX, 5–10 µM), and picrotoxin (100 µM) were added to the external solution listed above. The intracellular solution used for current clamp experiments contained (in mM): 135 KMeSO_3_, 10 HEPES, 4 MgCl_2_, 4 MgATP, 0.4 NaGTP, and 10 phosphocreatine. The intracellular solution used for voltage clamp experiments contained (in mM): 125 CsMeSO_3_, 20 HEPES, 4 MgCl_2_, 4 Mg_2_ATP, 0.4 NaGTP. ATP, GTP, 15 µM Alexa Fluor 594 and 300 µM Fluo-5F (Invitrogen, Carlsbad, CA) were added on the day of recording. Electrophysiological data were collected using custom software (J.S. Diamond, NINDS, Bethesda, MD) written in IgorPro (Wavemetrics,).

Recordings were performed at 32–34°C using an in-line heater (Warner Instruments, Hamden, CT). For the experiments presented in Figs. 2 and 3, before measuring intracellular Ca^2+^ transients, a series of voltage jumps (60–120 trials of 40 ms×70 mV) was used to run-down voltage-gated calcium channels (VGCCs) remaining in the presence of VGCC antagonists. The iontophoretic pipette contained 100 mM L-aspartate that was ejected by leak or a negative current (<−200 pA). Ejection was terminated by applying a positive backing current (1–2 nA).

### Two-photon imaging

Fluorescence was monitored with a custom-built 2PLSM using an Olympus upright microscope and objective (60×, 0.9/1.0 NA) and a Chameleon Ti:Sapphire laser (Coherent) tuned to 810 nm. Green and red fluorescence was collected by photomultipliers (H8224PA-40 or H10770PA-40, Hamamatsu) in both epi- and transfluorescence pathways using a 565 dichroic mirror and 525/50 and 620/60 band-pass filters (Chroma Technology). Images and line scans were acquired with ScanImage software [42].

### Data Analysis

Data analysis was performed using Image J, Microsoft Excel, Axograph X, and BrightStat. Student's t-test and ANOVA (Friedman with Conover post hoc) were used as noted. For older animals, the Ca^2+^ measurements in spines outnumber those of the dendrites due to several instances in which two spines were coplanar with the dendrite, allowing for simultaneous recording from all three structures. No spine was examined without its adjoining dendrite.

### Chemical sources

Drugs were obtained as follows: D-(-)-2-Amino-5-phosphonopentanoic acid (D-AP5), (R)-3-(2-Carboxypiperazin-4-yl)propyl-1-phosphonic acid (R-CPP), and NBQX, from Ascent Scientific; picrotoxin and VGCC blockers, mibefradil and nimodipine, from Sigma Aldrich; all other drugs from Tocris Bioscience.
